# Review of Recent Pharmacoepidemiologic Post-Market Safety Studies Through the Lens of the Estimand Framework

**DOI:** 10.1007/s43441-025-00780-4

**Published:** 2025-05-27

**Authors:** Yong Ma, Jonathan Haddad, Wei Liu, Ellen Snyder, Dimitri Bennett, Susan Mayo

**Affiliations:** 1 Office of Biostatistics, Office of Translational Sciences, Center for Drug Evaluation and Research, Food and Drug Administration, 10903 New Hampshire Avenue, Silver Spring, MD 20993 USA; 2 GlaxoSmithKline Plc, London, UK; 3 Office of Surveillance and Epidemiology, Center for Drug Evaluation and Research, Food and Drug Administration, Silver Spring, USA; 4 Merck & Co., Inc., Rahway, NJ USA; 5 Takeda Development Center Americas, Inc., Cambridge, MA USA; 6 University of Pennsylvania Perelman School of Medicine, Philadelphia, PA USA

**Keywords:** Estimand, Pharmacoepidemiologic study, Confounder, Intention-to-treat (ITT), Intercurrent event (ICE), Clinical trial emulation

## Abstract

The ICH E9(R1) estimand framework provides a systematic approach to ensure alignment among clinical trial objectives, trial conduct, statistical analyses, and interpretation of results, however, whether it can be readily utilized for the pharmacoepidemiologic safety studies has not been established. We selected articles on drug safety published in the Journal Pharmacoepidemiology and Drug Safety (PDS), during 2020 to investigate whether estimand attributes were well defined in the study design and reporting. We found that among twenty-five articles selected, nineteen were cohort studies and six were nested case-control studies. All studies had well-defined exposure, outcome, target population, and population level summary. The term intercurrent event (ICE) was not mentioned in any of the studies; however, many cohort studies discussed drug discontinuation, treatment modification and terminal events, and strategies to handle them. All studies used methods to control for confounding: propensity score methods or covariate adjustment, or both for cohort studies; matching and covariate adjustment for the nested case-control studies. We conclude that while the estimand framework can serve to add clarity and precision to pharmacoepidemiologic safety studies, more detailed considerations are required for bias assessment to compensate for the lack of randomization and other shortcomings in observational studies. Recent pharmacoepidemiology frameworks, such as Target Trial Emulation, STaRT-RWE, HARPER could be combined with the complementary principals from the estimand framework to help achieve the study objectives.

**Supplementary Information:**

The online version contains supplementary material available at 10.1007/s43441-025-00780-4.

## Introduction

**Fig. 1:**
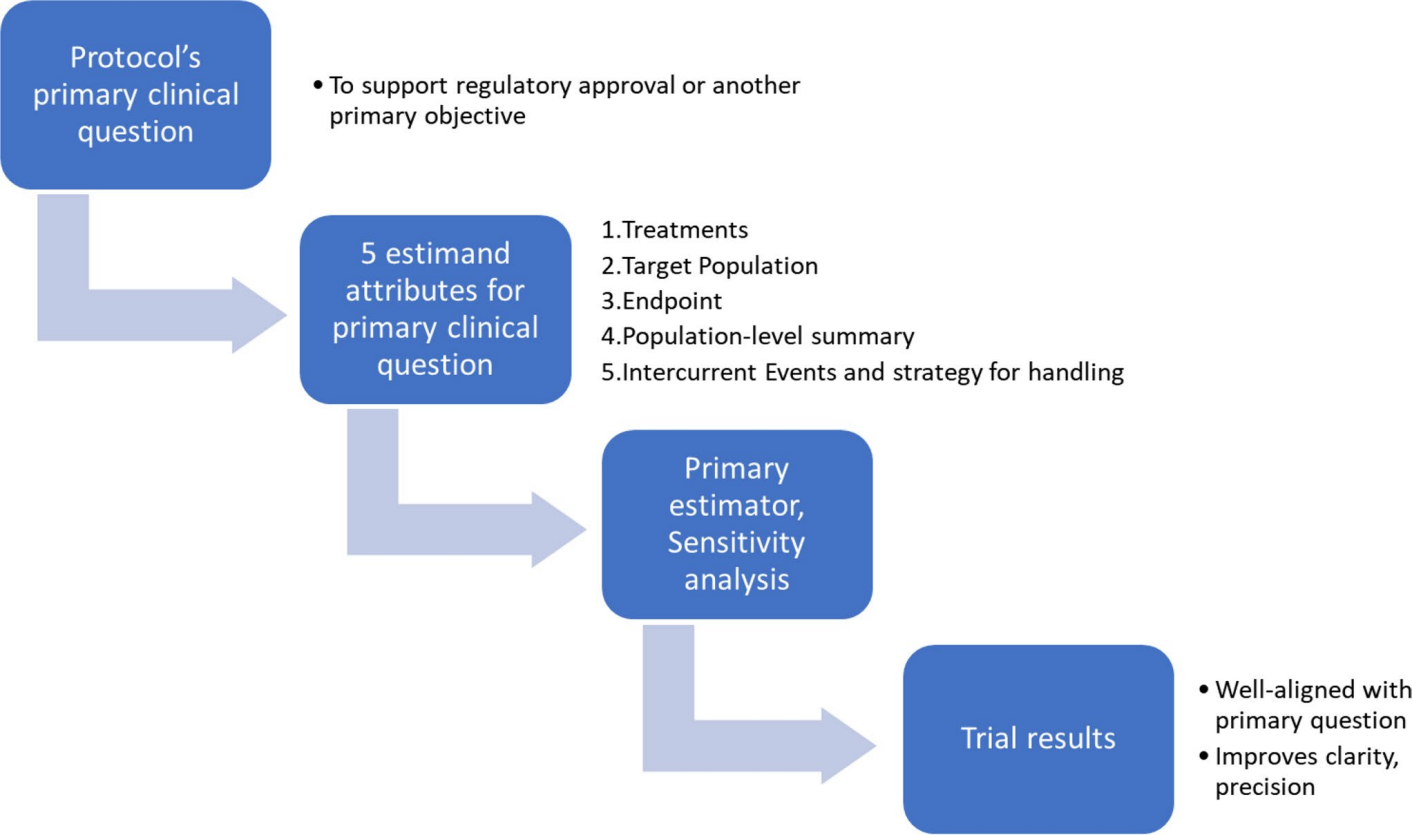
How the estimand is used in context with the primary clinical question of the study, the primary estimator, and the impact on trial results

The International Committee for Harmonization of Technical Requirements for Pharmaceuticals for Human Use (ICH) is a global international association that brings together regulatory authorities and pharmaceutical industry representatives to discuss scientific and technical aspects relevant to the development of highly effective and safe medicines [1]. In 2019, an addendum (R1) to the ICH E9 guideline on statistical principles for clinical trials introduced the estimand framework as a systematic approach to ensure alignment among clinical trial objectives, trial conduct, statistical analyses, and interpretation of results [2].This framework defines five key attributes as illustrated in Fig. 1:

1. **Treatment**: This refers to the treatment condition of interest and, if applicable, the alternative treatment condition for comparison. It can include combinations of interventions administered concurrently, such as add-ons to standard of care.
2. **Variable (endpoint)**: The variable or endpoint to be collected for each patient to address the clinical question.
3. **Population**: The population of patients targeted by the clinical question, which could represent the entire trial population or a subgroup defined by baseline characteristics.
4. **Intercurrent event (ICE)**: Events occurring after treatment initiation that impact the interpretation or the existence of the measurements related to the clinical question. Addressing ICEs helps precisely define the treatment effect to be estimated.
5. **Population-level summary**: A summary measure for the variable, serving as a basis for treatment comparisons, such as mean values at a specific timepoint, mean time to event, frequency, etc.

A key aspect of defining an estimand is recognizing ICEs and developing strategies to address them. The description of an estimand should reflect the clinical question of interest in respect to these intercurrent events and the choice of strategies to handle the ICE would provide additional clarity regarding the exact clinical question of interest that the study could answer. The ICHE9(R1) guidance outlines some strategies which may be used for handling them:

- **Treatment Policy**: Considering the occurrence of intercurrent event irrelevant in defining the treatment effect. The treatment effect, regardless of (or despite) an intercurrent events occurrence, is targeted.
- **Hypothetical**: Envisioning scenarios where the intercurrent event does not occur. The treatment effect in a particular hypothetical scenario is targeted.
- **Composite Variable**: Incorporating the intercurrent event into the variable definition (e.g., intercurrent event is assigned a particular value of the outcome variable).
- **While-on-Treatment**: focusing on treatment response before the intercurrent event.
- **Principal Stratum**: Concentrating on treatment effect within specific strata based on intercurrent event. (e.g., the subgroup of the population who would always adhere)

While ICH E9(R1) primarily pertains to randomized clinical trials (RCTs), the estimand concept is relevant whenever a treatment effect is estimated, or a hypothesis related to a treatment effect is tested, and the principles are applicable for observational studies [2, 3]. However, the widespread adaptation and utilization of estimand principles in pharmacoepidemiologic studies has yet to occur. Recently, regulatory agencies (FDA, EMA), and organizations like the International Society for Pharmacoepidemiology (ISPE) and the International Society for Pharmacoeconomics and Outcomes Research (ISPOR) provided recommendations and guidelines [4–6] on conducting pharmacoepidemiologic studies, emphasizing the importance of clearly defining key elements similar to estimand attributes that need to be specified in study protocols and reports. To assist investigators in developing observational study protocols for pharmacoepidemiology, ISPE and ISPOR jointly introduced STaRT-RWE [7] and HARPER [8] templates in 2021. These templates provide structured frameworks for documenting study parameters and scientific decisions that define the casual question. While the four main attributes of the estimand framework (population, treatment, variable of interest, and population-level summary) are commonly included in these templates, the operational details for each element may vary. Notably, the concept of ‘intercurrent event’ is not explicitly stated in these frameworks but is discussed in sections related to population, variable of interest, and treatment attributes. Furthermore, the European Network of Centres for Pharmacoepidemiology and Pharmacovigilance (ENCePP) published their 11th Revision on methodological standards in July 2023 [^9^] and recommended considering the estimand framework to inform study design and data analysis choices. However, the details on how to align the five estimand attributes to pharmacoepidemiologic studies are not discussed in this standard. In addition, the target trial emulation, originally proposed by Hernan and Robins [10], is another causal framework developed to help answer causal questions by applying design principles from randomized trials to the analysis of observational data.

**Fig. 2:**
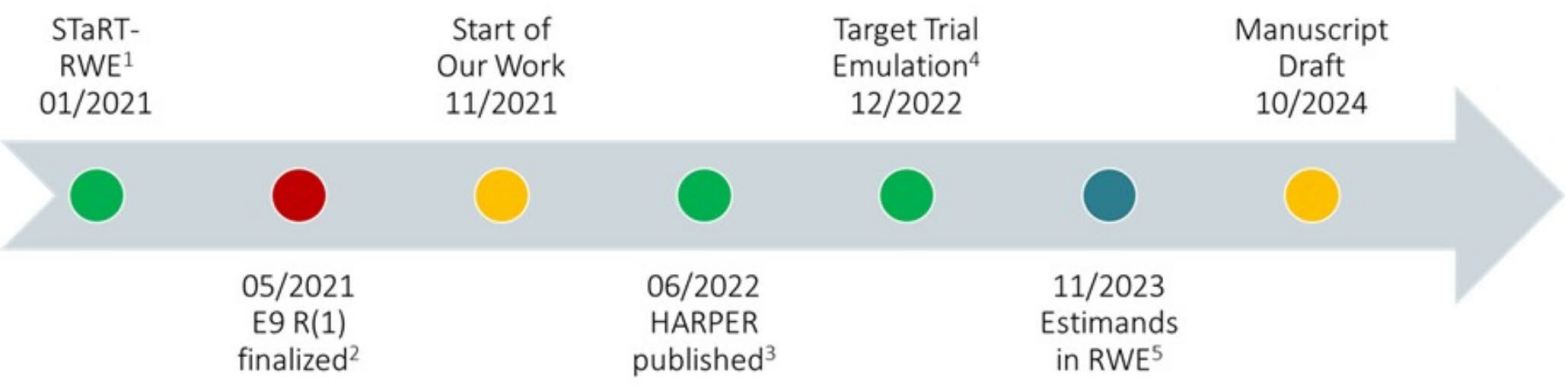
Timeline of our work in relation to the publication of estimand framework and relevant RWE frameworks. (1).Publicationof STaRT-RWE: structured template for planning and reporting on the implementationof real-world evidence studies in BMJ [7]. (2). Publicationof the estimand framework: E9(R1) [2]. (3). Publicationof HARmonized Protocol Template to Enhance Reproducibility of HypothesisEvaluating Real-World Evidence Studies on Treatment Effects: A Good PracticesReport of a Joint ISPE/ISPOR Task Force in Value Health [8]. (4). Publicationof Target Trial Emulation: A Framework for Causal Inference FromObservational Data in JAMA [10]. (5). Publicationof Estimands in Real-World Evidence Studies. Statistics in Biopharmaceutical Research [36]

In this article, we seek to assess the contemporary practice in design and analysis of non-randomized post-marketing safety studies through the lens of the estimand framework. Although the terminology in these studies may vary, we suspect that many of the underlying concepts in estimand may have already been utilized. This article aims to focus on identifying the estimand attributes in recently published pharmacoepidemiologic studies. Figure 2 highlights the timeline of our study in relation to the publication of the estimand and other pharmacoepidemiologic guidelines.

## Methods

Post-market safety observational studies were searched in the journal *Pharmacoepidemiology and Drug Safety (PDS)*, and studies meeting the pre-specified selection criteria, described in the next paragraph, were selected. Information related to the five estimand attribute fields was then extracted. We selected *PDS* because it is the official journal of the International Society of Pharmacoepidemiology (ISPE), providing access to numerous relevant studies and featuring contributions from authors and reviewers knowledgeable about recent methodological advances.

The search criteria restricted inclusion to studies published in the year 2020 as “Original Reports” or “Original Articles”. These studies had to be observational, including cohort studies, case-control, nested case control studies or case crossover studies. They also had to address safety questions in the post-marketing setting regarding treatment effects, e.g., studies addressing only efficacy endpoints were excluded. Type of studies that did not meet the above criteria include those that focused on: descriptive analysis, methods to evaluate algorithms, data source description or assessment, statistical methods, policy evaluation, and cross-sectional studies, pharmacovigilance studies, and review studies.

Two study team members (WL, YM) together reviewed all the articles published digitally in PDS in 2020. Twenty-five articles were selected based on the specified criteria. Each study team member read 5–6 articles and identified the use of estimand attributes, entering them into a database (excel file). Information concerning the estimand framework attributes “treatment (often referred to as exposure)”, “variable (often referred to as outcome)” and “population” appeared consistently in “Materials and Methods” section of the articles, and information about “population-level summary” was found in the “Analysis” or “Statistical Analysis” sections.

Because the term “intercurrent event” was not used in any of the studies, the authors considered for inclusion of the following events as ICEs: medication discontinuation, medication modification such as the use of additional medication or alternative medication, and terminal events such as death. These ICEs were mentioned in varying sections of the article.

## Results

Among the 25 selected studies [11–35], 19 were cohort studies, and 6 were nested case-control studies. Within the 6 nested case-control studies, one was conducted jointly with a case-crossover study [13] and another with a case-time-control study [16]. Based on our study selection criteria, all of these studies aimed to identify a causal association between drug exposure and safety outcomes.

Table 1 summarizes of the characteristics of the cohort studies, while detailed information for each article can be found in supplemental Table 1A, B. Seven studies were conducted in the United States, and 12 outside the United States. Data sources included claims (*N* = 3), electronic health record (EHR) (*N* = 2), registries (*N* = 3), linked EHR and claims (*N* = 8), and longitudinal studies with primary data collections other than registries (*N* = 3). Three studies were prospective, and 16 were retrospective. Methods used for confounder control included propensity score (PS) methods (*N* = 7), covariate adjustment through modeling (*N* = 9), the use of both PS and modeling (*N* = 2) or exact matching (*N* = 1). Statistical methods used for primary analysis included time-to-event analysis, such as Cox’s proportional hazards (PH) modeling or using the Aalen-Johansen estimator (*N* = 10), logistic regression, generalized estimating equation (GEE) or Chi-square test (*N* = 6), as well Poisson regression (*N* = 3).

**Table 1:** Summary of selected characteristics and analysis methods for the cohort studies

Study Characteristics	Total (*N* = 19)	%
Region		
US	7 (20,23,25,26,31–33)	37%
Non-US	12 (17–19,21,22,24,27–30,34,35)	63%
Data sources		
Claims	3 (23,25,33)	16%
Electronic Health Records (EHR)	2 (20,28)	11%
Registry	3 (18,29,30)	16%
EHR and claims linked	8 (17,19,21,22,27,31,32,35)	42%
Longitudinal study	3 (24,26,34)	16%
Study type		
Prospective	3 (24,26,34)	16%
Retrospective	16 (17–23,25,27–33,35)	84%
Methods for confounder control		
Propensity Score (PS) method	7 (21–23,25,28,30,35)	37%
Covariate adjustment through modeling	9 (17,18,24,26,27,29,31,33,34)	47%
Both PS and modeling	2 (19,20)	11%
Exact matching	1 (32)	5%
Statistical Modeling for primary analysis		
Time to event (Cox’s Proportional Hazard model/Aalen-Johansen estimator)	10 (19–23,26–29,31)	53%
Logistic regression/GEE/Chi-square test	6 (18,24,25,33–35)	32%
Poisson regression	3 (17,30,32)	16%

*Article reference numbers are enclosed in parentheses

All studies included sections describing exposure, study outcome, and the statistical methods used to obtain summary measures and the details are described in supplemental Table 1A, B. We would like to highlight several key features of these cohort studies. The study population was generally broad, a few population-based, with few exclusion criteria, and included patients from broad demographic backgrounds. The exposure involved complex treatment patterns, with some therapies frequently discontinued or modified, and variations in timing and duration, reflecting real-world clinical practice. The outcome was often captured using algorithms or rule-based approaches, with outcome data typically collected irregularly, sometimes under-reported, and subject to information bias. Full or partial adjudication, or validation of existing algorithms was usually included. The population-level summary approaches were similar to those used in RCT, and all studies included methods for confounder control. Since the term “ICE” was not explicitly used in the studies, we provide a detailed summary of ICEs descriptions and strategies, organized by their location within the articles (Table 2) and the types of strategies employed (Table 3).

**Table 2:** Article sections where intercurrent events (ICEs) were identified for the cohort studies

Article Sections	Total (*N* = 16)	%
Analysis	8	50%
Primary Analysis	1 (20)	
Sensitivity Analysis	2 (21,23)	
Statistical Analysis	5 (17,18,26,30,31)	
Exposure	4	25%
Exposure	2 (25,29)	
Exposure and covariates	1 (22)	
Medication exposure and analysis populations	1 (27)	
Study population	3	19%
Study population	2 (19,22)	
Study population, design and data source	1 (23)	
Outcome	2	13%
Study outcome and endpoints	1 (19)	
Outcome and cohort followup	1 (28)	
Follow-up	2	13%
Follow-up	1 (21)	
Follow-up time	1 (20)	
Results	2 (18,32)	13%
Study design	1 (32)	6%
Discussion	1 (33)	6%

*Article reference numbers are enclosed in parentheses. Studies 24, 34 and 35 reported no intercurrent event and are not included in the denominator for the percentage calculations. Also, counts are not mutually exclusive as one article could have reported more than one intercurrent event and they could appear in multiple sections

**Table 3:** Categorization of intercurrent events and strategies used for the cohort studies

ICE	ICE Strategies	Total (*N* = 16)	%
Medication (Exposure) discontinuation	While-on-treatment 6 (19,21,22,25,27,28) Treatment policy 2 (18,25,27) Others Comparison* 1 (18), Time-varying exposure 1 (27)	7 (18,19,21,22,25,27,28)	44%
Medication modification (Additional/Alternative)	Treatment policy 6 (19,20,21,27,29,32) While-on-treatment 5 (21,22,25,27,28) Hypothetical Exclusion 2 (31,33) IPCW** 1 (25) Others Secondary endpoint* 1 (19) Time-varying exposure 1 (27)	11 (19–22,25,27–29,31–33)	69%
Terminal Events	While alive*** 9 (18,20,26–32) Hypothetical Exclusion 3 (21,23,33) Down weighting 1 (17) Competing risk analysis 1 (25)	14 (17,18,20,21,23,25–33)	88%

*Some studies performed additional analysis to investigate the effect of drug discontinuation on the primary outcome

**IPCW - inverse probability of censoring weighting

*** While alive is considered a form of While-on-treatment (See E9(R1) for more details)

Sixteen out of the 19 cohort studies (84%) reported ICE events. These ICE events were found in various sections of the articles and are summarized in Table 2. The analysis section appeared to be the most frequent place for addressing ICEs, with 50% of the articles (*N* = 8) utilizing this section to describe ICEs and strategies to handle them. Exposure ranked as the second most frequent section, appearing in 25% of the articles (*N* = 4), followed by the study population section at 19% (*N* = 3). Although less commonly, ICEs and related strategies were also found in sections such as outcome, follow-up, study design, results, and discussion.

Table 3 summarizes the specific ICEs and the strategies used to address them. Seven articles addressed medication discontinuation, while 11 articles discussed other treatment modifications, including the addition of other treatments, the use of alternative treatments, or treatment switching. Fourteen articles addressed terminal events like death. Regarding strategies for addressing medication discontinuation, 6 out of 7 articles used while-on-treatment strategy, 2 employed treatment policy methods. Other analysis not belonging to the five ICE strategies specified in E9 (R1) were also used; one compared the effect on the outcome between the discontinued group and the non-discontinued group, and another defined exposure as a time-varying covariate.

For medication modifications, 6 articles used treatment policy strategy, 5 articles used while-on-treatment strategy, and three articles used hypothetical strategies. The hypothetical strategies included two different scenarios envisaged. The first is exclusion, assuming those with medication modifications behaved the same as those not having medication modifications. The second is using inverse probability of censoring weights (IPCW), where censoring is considered as a random event not associated with the outcome. Other analyses included one treating the modification as a secondary endpoint, and another using time-varying exposure. In the case of terminal events, 9 articles used while-on-treatment, also termed “while-alive” in this scenario. Hypothetical strategy is used for 5 articles, where 3 used exclusions, one used down-weighting, and one utilized competing risks analysis, each with its own underlying hypothesis. It is also common that a study may use different ICE strategies to address different ICEs.

There were 6 nested case-control studies, all conducted outside the United States. Three of these studies used EHR data, and other three used claims data. Due to the small number of case-crossover (*N* = 1) and case-time-control (*N* = 1) studies, these two are not described further in this article. Details of the nested case-control studies are described in supplemental Table 2A, while cohort definition, exposure details, and terminal events are described in supplemental Table 2B.

Although nested case-control studies originated from cohort studies, the study design identifies the outcome (case) first, matching the case with people who had no event, and then examine their exposure status. All six studies utilized matching with varying matching ratios, conducted conditional logistic regression for analysis and reported odds ratio (OR) as the population level summary. Many studies emphasized on the dose and timing of exposure. Five studies [11–15] considered treatment timing or duration to examine the timing (dose) -response relationship. The only exception was when the exposure involved a very short treatment regimen lasting only 7 days [16].

Regarding ICE and strategies, the nested case-control studies are different. The index date is defined as the time of outcome occurrence of case patients, and this resulted in two issues. First, terminal events before the occurrence of the event of interest prevent the observation of a case, hence those who experienced terminal events could only be included in the pool of potential controls. Unless the original cohort was clearly defined, ICE of terminal events may not be identified and handled properly. Among the six studies, two studies [12, 15] clearly defined a follow-up period for the study cohort, during which ICEs such as terminal events were censored. However, the four remaining studies only described cohort entry without specifying the follow-up process, not addressing the potential ICE of terminal events. Secondly, many of the six studies didn’t distinguish between confounding factors occurring before or after the drug exposure. Rather, a distinct time period before the index date was used to capture confounders, similar to how exposure was captured in a time period before the index date. Covariates captured in this manner included a mix of variables prior or after the exposure, and the analyses treated both types equally. As a result, ICE such as additional medication, medication modification cannot be identified as these need to happen after the exposure. Considering the challenges inherent in the study design, it may be challenging to discern the concept of ICE in case-control studies.

## Discussion

We identified and reviewed 25 inferential pharmaco-epidemiologic studies addressing safety issue(s) that were published in PDS in 2020 through the lens of the estimand framework. Of these, 6 were case-control, and 19 were cohort studies and all of which had clearly defined exposure, outcome, target population, and population level summary. The term “intercurrent event” was not explicitly mentioned, however, many articles reported events such as medication discontinuation, modification, or terminal events, along with strategies to handle them. These events and strategies were found in various sections of the articles. For cohort studies, while-on-treatment and treatment policy strategy were the most frequently used to handle modifications, additions and discontinuation of treatment. Hypothetical strategies such as censoring or exclusion were commonly used to handle terminal events. For case-control studies, exposure was often categorized according to the length or timing of exposure. All studies used strategies to control for potential confounding by baseline factors.

While our study is focused on the use of 5 attributes of estimand framework, we want to note that the study questions specified in each of the articles we reviewed are typically general in nature and do not mention information about intercurrent events or other estimand attributes. Although a general study question may be helpful at the beginning of the study planning, the study questions can be further clarified by consideration and specification of the estimand attributes. One limitation of our study is that we had no access to study protocol or the statistical analysis plans and therefore won’t be able to see if a more specific scientific question was constructed in these studies.

**Fig. 3:**
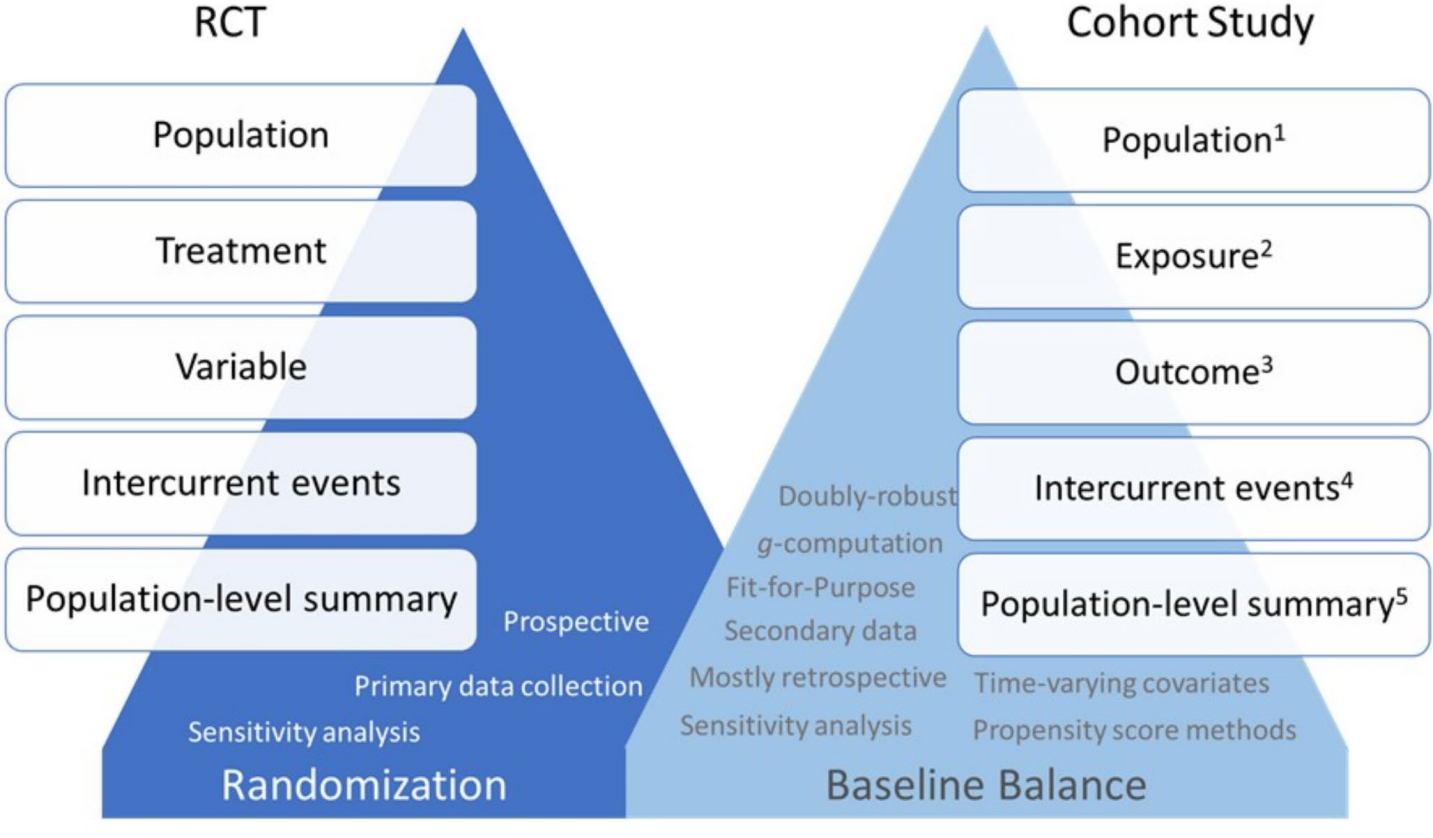
Similarity of estimand attributes in clinical trials and pharmacoepidemiologic cohort studies. (1). Populationin a cohort study is typically more heterogeneous, including patients with morediverse demographic backgrounds. (2). Exposurein a cohort study may have more complex treatment patterns, reflecting realclinical practice. (3). Outcomein a cohort study depends on both the research question and availability of RWDdata sources. The collection of outcome data could be irregular,under-reported, and subject to information bias. Full or partial adjudication,or validation of an existing algorithm is usually required. (4). Intercurrentevents in a cohort study may be multiple with various approaches to addressthem. Due to the secondary nature of RWD in most cohort studies, some ICEs maynot be captured. (5). Justlike in an RCT, population-level summary in cohort studies depends on thedefinition of the outcome and the research question

The estimand framework, designed for clinical trials, also captures core attributes of post-marketing pharmacoepidemiologic studies, as demonstrated by the relatively consistent reporting of treatment, population, variable and population level summary attributes in studies reviewed. Figure 3 provides a rough summary of the alignment between the estimand framework for RCT and pharmacoepidemiologic cohort studies. In the clinical trial setting, data are collected prospectively and are well controlled. In contrast, the pharmacoepidemiologic studies are more likely to be retrospective and uncontrolled, and the data sources are often secondary such as coming from claims, EHRs, and registries. Thus, data sources characterization (purpose, collection methods, fit-for-purpose) should be explicitly described. As a result, the operational definition of the aforementioned four attributes is more complex than the definition used in clinical trials. For example, in clinical trials, the start of follow-up is defined by the time of randomization, while determining ‘time-zero’ for participants in nonexperimental studies is more complex. This complexity can lead to issues like immortal person-time, arising from the misalignment between time of eligibility and treatment initiation [36]. A three-step procedure - cloning, censoring, and weighting - that emulates the analysis of randomized trials with full adherence helps eliminate immortal time bias [37]. Another major issue inherent in pharmacoepidemiologic study design is selection bias, which occurs due to insufficient adjustment for pre- and post-baseline prognostic factors which may influence both medication adherence or lost to follow-up, and ultimately, outcome capture [38]. Both issues can be particularly severe when outcome risks change over time. For instance, in comparative safety studies of anticancer drugs where patients could have received multiple lines of eligible therapy before entering either cohort, an imbalance in prior lines of therapy between treatment groups can introduce bias, affecting the study’s validity.

Successful randomization in clinical trials effectively removes measured and unmeasured confounding, a challenge frequently encountered in pharmacoepidemiologic studies where systematic differences among treatment groups may arise. In pharmacoepidemiologic studies, it is crucial to implement confounding control through thoughtful study design and/or prespecified statistical analysis. Additionally, prespecified qualitative and quantitative methods, such as quantitative bias analysis, are essential to evaluate the impact of residual confounding. The articles reviewed showed that both cohort and case-control studies addressed confounding concerns through statistical modeling methods such as propensity score techniques, covariate adjustment, or a combination of both.

Although ICE is a new term and it was not used in any of the studies, many studies discussed the handling and impact of ICEs. For cohort studies, introducing the concept of ICE may distinguish between baseline covariates and time-varying covariates, clarifying the distinct roles each type of covariate plays. The baseline covariates must be balanced to emulate the effect of randomization. The use of time-varying covariate methods helps address confounding that may arise after the index date due to ICEs. The distinction of confounding arising from baseline imbalances or ICEs is not a concern for the nested case-control studies, as both would be analyzed in the same manner, and making such a distinction unnecessary. Instead, in the context of the nested case-control study, it is important to note that the study originates from a cohort and preferably the cohort can be explicitly defined using the estimand framework.

The ICH E9(R1) estimand framework emphasizes the consideration of ICEs, which may be reflected in population, variable, or treatment attributes. Similarly, in the articles we reviewed, ICEs appeared in various sections such as study population, exposure, outcome, in addition to statistical analysis. Compared to clinical trials, in pharmacoepidemiologic studies, treatment regimen is usually more complex; adherence and persistence could differ markedly; and outcomes are usually not captured in the same rigorous way as randomized trials. Thus, ICEs may be more prevalent in pharmacoepidemiologic studies. In addition, it is not uncommon that a large proportion of data such as laboratory tests, vital signs, or over-the-counter medication are missing or incompletely captured from the data source, and such uncaptured data could lead to underreporting of ICEs and their appropriate data handling strategy in pharmacoepidemiologic studies.

We recognize that ICEs may be addressed in different ways in pharmacoepidemiologic studies. For example, using the treatment policy approach for ICEs in efficacy studies would be considered conservative, as the occurrence of ICEs could lead to a reduced estimate of the magnitude of drug effectiveness. However, this strategy may not be preferable in evaluating safety endpoints, as it could underestimate a potentially important safety signal. This consideration may be the key factor driving the preference for the while-on-treatment strategy when assessing treatment discontinuation in the articles reviewed. Note that while the articles we reviewed used “intent-to-treat” to refer to the “treatment policy” approach for handling medication discontinuation or switching, the two terms are not synonymous. Intent-to-treat means that participants in a clinical trial are analyzed in the group to which they were originally randomized, regardless of whether they completed or adhered to the assigned treatment. For example, the intent-to-treat approach includes scenarios where subjects receive the incorrect treatment but are still analyzed in the group to which they were randomized, extending beyond events occurring after randomization. Nevertheless, regardless of whether the treatment policy or while-on-treatment approach is used, confounding and selection bias may arise and must be addressed, as described by Hernán et al. [39]. It’s worth noting that the reviewed articles exhibited variability in their approach to describing ICEs and the rigor of their strategies. Some studies meticulously characterized ICEs, while others place less or no emphasis on these occurrences. Let’s spotlight two studies at opposite ends of the spectrum. One article [25] described in depth how medication discontinuation or switching was handled and compared the impact of on-treatment (OT) or initial treatment (IT) strategies, equivalent to the while-on-treatment and treatment policy strategies, respectively. For certain outcomes, the two approaches yielded results in opposite directions and for other outcomes, they led to attenuated treatment effects. The study attributed this observation to a significantly higher proportion of study participants discontinuing in one study arm compared to the other. This observation underscores the unignorable impact of ICE strategies on the study results. Conversely, in the Swedish Registry study of Sekunimab vs. Ustekunimab [29], the investigators used an ‘ever|never’ approach to assigning treatment arm, analyzing safety outcomes by the initial treatment given, irrespective of treatment switching even though there was a considerable level of switching (~ 12%). The Investigators didn’t perform any analyses to evaluate the impact of switching. It also brought to our attention that ICE was not simply viewed as an event where it may impact the safety outcomes. At least in two studies [18, 19], medication discontinuation or modification was analyzed as a study endpoint, providing insight into the practical use of these medications.

Since the publication of ICH E9(R1), the estimand framework has been evaluated by clinicians, statisticians, and epidemiologists, exploring whether it could be used beyond the clinical trial setting. Careful consideration and a roadmap for choosing appropriate estimand for pharmacoepidemiologic studies have been proposed [40]. The estimand framework and the epidemiologic frameworks were developed by distinctive communities for different purposes, i.e., estimand framework for clinical trials; Start-RWE, HARPER, and Target Trial Emulation for observational studies. Both estimand and epidemiologic frameworks could be used simultaneously to benefit the design of pharmacoepidemiologic studies. Recently, researchers applied this approach in two oncology RWE studies leveraging RWD to construct external control arms to supplement single-arm trials that investigate the efficacy and safety of oncology drugs [41, 42].

This current work has some limitations. First, we focused on treatment discontinuation, use of alternative therapies, and terminal events. However, there could be other ICEs that can also occur in real-world studies. For example, a patient initiating over-the-counter medication, or the frequencies of visit schedules, insurance or hospital policy regarding the allowed concomitant medications, or the learning effects associated with a test as the patient becomes familiar with the test procedure. Retrieving articles only from PDS may have also introduced some bias because of the focus of articles published in this specific journal.

## Conclusion

In conclusion, this research demonstrated that the key design elements in contemporary pharmacoepidemiologic studies can be identified through the lens of the ICH E9(R1) estimand framework. However, because non-randomized studies are inherently complex, pharmacoepidemiologic research involves a wider range of variations and challenges, as evidenced by the extensive and detailed frameworks such as STaRT-RWE, HARPER, and Target Trial Emulation. While the estimand framework provides a general principle for causal inference, a well-defined plan that incorporates these details through pharmacoepidemiologic frameworks would improve transparency, clarity, and the overall execution of study objectives.

## Electronic Supplementary Material

Below is the link to the electronic supplementary material.

Supplementary Material 1

## Data Availability

No datasets were generated or analysed during the current study.
