# Correction: Review of Recent Pharmacoepidemiologic Post-Market Safety Studies Through the Lens of the Estimand Framework

**DOI:** 10.1007/s43441-025-00821-y

**Published:** 2025-06-14

**Authors:** Yong Ma, Jonathan Haddad, Wei Liu, Ellen Snyder, Dimitri Bennett, Susan Mayo

**Affiliations:** 1 Office of Biostatistics, Office of Translational Sciences, Center for Drug Evaluation and Research, Food and Drug Administration, 10903 New Hampshire Avenue, Silver Spring, MD 20993 USA; 2 GlaxoSmithKline Plc, London, UK; 3 Office of Surveillance and Epidemiology, Center for Drug Evaluation and Research, Food and Drug Administration, Silver Spring, USA; 4 Merck & Co., Inc, Rahway, NJ USA; 5 Takeda Development Center Americas, Inc, Cambridge, MA USA; 6 University of Pennsylvania Perelman School of Medicine, Philadelphia, PA USA

**Therapeutic Innovation & Regulatory Science**

10.1007/s43441-025-00780-4

A disclaimer was missing from the Declarations section: “This paper reflects the views of the authors and should not be construed to represent the views or policies of the FDA or the authors’ employers.”

