# Supplementary material for: Review of Recent Pharmacoepidemiologic Post-Market Safety Studies Through the Lens of the Estimand Framework
Source: Ther Innov Regul Sci. 2025 May 27;59(5):971–80. doi: 10.1007/s43441-025-00780-4 (PMC12446131; doi:10.1007/s43441-025-00780-4)
Supplement: Supplementary file 1 — Supplementary Material 1 [file 43441_2025_780_MOESM1_ESM.docx]

Supplemental Table 1A. Cohort studies and features of estimand attributes including exposure, target population, outcome and population level summary.

| **Reference** | **Study design** | **Study Question** | **Data Type/source** | **Exposure** | **Target population of interest** | **Outcome measure of interest** | **Population level Summary** |
| --- | --- | --- | --- | --- | --- | --- | --- |
| W. Umek^16^ | Retrospective | To investigate the role of prescription drugs in the etiology of the overactive bladder (OAB). | Population-wide comprehensive data, Austria | use of anticholinergic medication and 188 chemical substances, which are suspected triggers for OAB (trigger medications [TMs]) in the 1^st^ half of 2012 | OAB-naïve women | Prescription for anticholinergic medication against OAB in the second half of 2012 | Relative Risk from Poisson regression |
| Gabriella Bröms^17^ | Retrospective, Population-based | To study the risk of preterm birth, caesarean section, and small for gestational age after anti-tumor necrosis factor agent treatment (anti-TNF) in pregnancy | National health registers in Denmark, Finland and Sweden | Anti-tumor necrosis factor agent treatment (anti-TNF) vs. non-biologic systematic treatment | Women with inflammatory bowel disease, rheumatoid arthritis, ankylosing spondylitis, psoriatic arthritis, and psoriasis, and their infants | Birth outcomes including: pre-term birth, small for gestational age and caesarean section | Odds Ratio |
| U. Gungabissoon^18^ | retrospective | To estimate the risk of dyskinesia and impulse control disorders (ICDs) in patients with Parkinson's disease (PD) prescribed ropinirole prolonged-release (R-PR) compared to those prescribed immediate-release dopamine agonists (IR-DA) as monotherapy | Clinical Practice Research Datalink (CPRD), UK | ropinirole prolonged-release R-PR or immediate-release dopamine agonists (IR-DA) | patients with Parkinson’s disease (PD) | dyskinesia and impulse control disorders (ICDs) | Incidence Rate Ratio, Hazard ratio |
| J Salas^19^ | Retrospective | If early initiation of metformin treatment is associated with a decreased risk of developing dementia compared with not initiating or delaying treatment. | 2 EHR databases: Veteran Health Affairs and Kaiser Permanente Washington, US | Metformin use (initiation vs. non-initiation, overall and stratified by baseline HbA1c, age, or race) | Patients ≥50 years with HbA1c value which made them qualified for metformin treatment | Dementia | Hazard Ratio |
| Y Xu^20^ | Retrospective | To evaluate the risk of heart failure with sulfonylurea use | Yinzhou Regional Health Care Database (YRHCD), an integrated EHR, health check, death data, China | Sulphonylureas vs. acarbose monotherapy | Type 2 diabetic patients ≥ 18 years | hospitalization for heart failure | Hazard Ratio |
| T Chyou^21^ | retrospective | To evaluate the association between incident Parkinsonism and type and dose of second-generation antipsychotic use | Nationwide inpatient hospital data named National Minimum Data Set (NMDS), New Zealand | Atypical antipsychotic (olanzapine, risperidone or quetiapine with quetiapine as the reference) | new users of second-generation antipsychotics (SGAs) in adults ≥65 years | Parkinsonism | Hazard Ratio |
| T Thai^22^ | Retrospective | To evaluate the risk of pregnancy loss and congenital malformations associated with first trimester exposure to mycophenolate vs. azathioprine use. | BM MarketScan Research Database, Claims database US | Mycophenolate (MPA) exposure compared to azathioprine (AZA) use; Using varying exposure assessment windows | Women 12-55 years with ≥1 MPA or AZA prescription claim during their first trimester | Pregnancy loss, congenital malformations | Relative risk, risk difference |
| E Skovlund^23^ | Prospective, population-based | To assess the effect of exposure to analgesic opioids during pregnancy on development of language and communication skills in 5-year old children | Population-based Mother and Child Cohort Study (MoBa), Norway | Maternal analgesic opioids exposure in 4 different ways 1) Yes/no; 2) during one period verse during two or all here pregnancy periods 3) repeated use vs occasional use and no use 4) stratified by trimeters. | 5 year old children from MoBa cohort study who had fetal exposure to opiods, 1999-2008. | Children's language competence and communication skills at 5 years | Odds Ratio |
| M Webster-Clark^24^ | Retrospective | to estimate risk differences for ischemic stroke, death, and gastrointestinal bleeding after initiating dabigatran and warfarin inolder adults (a) when patients adhere to treatment and (b) under real-world adher-ence patterns. | Medicare Claims database, US | Dabigatran or warfarin use | adults over age 65 | ischemic stroke, death and gastrointestinal bleeding, analyzed separately | Risk difference |
| B Harding^25^ | Prospective | evaluated whether these medication swere associated with incident clinically detected atrial fibrillation (AF) or monitor-detected supraventricular ectopy (SVE), including premature atrial contractions (PACs)and supraventricular tachycardia (SVT) | Multi-Ethnic Study of Atherosclerosis (MESA) cohort, US | time-varying exposure to (a) opioid, (b) gabapentinoid or (c) NSAID medications at the first five exams (Exams 1-5) compared with nonusers. | Adults 45-84 years old who were free of clinically recognized cardiovascular disease | incident clinically detected atrial fibrillation (AF) as the longitudinal; other cross-sectional outcomes not included for this table | Hazard Ratio, |
| T Althunian^26^ | Retrospective | To compare the effectiveness and safety of a drug in daily practice with the outcomes of a target non-inferiority trial by rigorously mimicking in an observational study the trial's design features | British Clinical Practice Research Datalink (CPRD), UK | Rivaroxaban exposure or warfarin use | Patients with nonvalvular atrial fibrillation (AF) | Major bleeding as the safety endpoint | Hazard Ratio |
| L. Fanning^27^ | population-based | Investigated gastrointestinal bleeding (GIB) risk with rivaroxaban vs aspirin among two separate AF cohorts in Hong Kong and the United Kingdom, using a common protocol approach | Clinical Data Analysis and Reporting System (CDARS) of the Hong Kong Hospital Authority and The Health Improvement Network (THIN) UK | Rivaroxaban vs aspirin | Patients aged ≥18 years with a new atrial fibrillation | gastrointestinal bleeding which included peptic, duodenal, gastrojejunal ulcers with haemorrhage, bleeding gastritis or duodentitis, gastrointestinal haemorrhage and intestinal haemorrhage | Hazard Ratio |
| C Srinivas^28^ | population-based register-linked | To determine risk of respiratory tract infections, urinary tract infections and candidiasis in secukinumab users compared to ustekinumab users among individuals with psoriasis in Sweden | Multiple national registers - National Patient Register (NPR), Swedish Prescribed Drug Register (PDR), Swedish Cancer Register (SCR), Cause of Death Register (CDR), and population registers in Sweden, Sweden | Secukinumab vs. ustekinumab | individuals with psoriasis and psoriasis arthritis | Common infections comprised of upper and lower RTI, UTI and candidiasis | IRR, Hazard Ratio |
| M Mahic^29^ | population-based | To study the impact of in utero opioid exposure on the immune system and the risk of infections in childhood | national health registers in Norway, Sweden, and Denmark | opioid maintenance therapy (OMT) or treatment of pain; Varying time and length of exposure grouped into four mutually exclusive groups | pregnant women and their children prenatally exposed to OMT opioids | infections in children until maximum 12 years of age | IRR using Poisson regression; HR from Cox’s PH |
| D Selig^30^ | retrospective | To explore patterns of antimuscarinic medication as a risk factor for type  2 diabetes mellitus (T2DM). | Pharmacovigilance Defense Application System (PVDAS)/Military Health System (MHS), US | Antimuscarinic medication | Females 18 years of age or older treated with an antimuscarinic medication | Type 2 diabetes mellitus for the overall population, by duration of therapy and by individual drugs | Hazard Ratio |
| A Gilsenan^31^ | population-based comparative | This study compared the incidence rate of  osteosarcoma among patients aged ≥65 years treated with teriparatide vs a  matched-comparator cohort. | Medicare linked to participating state cancer registry data, US | Teriparatide vs. other medication that is not to treat osteoporosis | Women 65 years or older | Incident Osteosarcoma | IRR from Poisson regression |
| D Barthold^32^ | Retrospective | Compared ADRD risk for users of non-selective and M3-selective  BAMs and examines ADRD risk associated with overall BAM use. | Medicare claims data, US | non-selective and M3-selective bladder antimuscarinics (BAMs) measured according to their total standardized daily doses (TSDD), which is calculated as an individual's total dose for a year, divided by the minimum daily dose that is specific to the efficacy of that drug | Individual at least 67 years old | Alzheimer's disease and related dementias | Odds Ratio |
| L Pasina^33^ | Prospective, door-to-door population-based | To assess the relations  between cognitive performance and ACB scores according to the hypothesis that  a higher anticholinergic burden is associated with reduced cognitive performance. | The Monzino 80-plus study database, Italy | anticholinergic medications | aged 80 years and older | cognitive impairment (Dementia and mini-mental state examination (MMSE) score) | Odds Ratio |
| D Sato^34^ | retrospective | To compare in-hospital mortality  and morbidity between short-term, low-dose quetiapine and risperidone in postoperative  patients with diabetes. | Diagnosis Procedure Combination (DPC) database, Japan | oral quetiapine or oral risperidone use | patients with diabetes aged 20 years or older who had undergone scheduled elective surgery | Primary outcome was in-hospital mortality | Relative Risk |

NA: Not Assessed

* Trial replication

Supplemental Table 1B. Cohort studies and features of ICE and ICE strategies and attributes.

| **Reference** | **ICE** | **ICE strategy** | **Section ICE event/strategy was mentioned** | **Confounder**  **control** |
| --- | --- | --- | --- | --- |
| W. Umek^16^ | Death | Women who died were downweighted in the regression model according to their proportional time at risk | Statistical analysis | Covariate adjustment for age, indicator of hospitalization and others |
| Gabriella Bröms^17^ | 1) Medication discontinuation; 2) stillbirth | 1) Compared continuation vs discontinuation of study medication; 2) no stillbirth in the treatment arm and no information given for the control arm and ignored in analysis | 1) Statistical analysis 2) results | Covariate adjustment in logistic regression |
| U. Gungabissoon^18^ | 1) Drug discontinuation and 2) initiation of alternative medication | 1) follow-up ended at treatment discontinuation plus 30 days. 2) Used treatment adherence and persistence and time to levodopa initiation as secondary endpoints. | 1) Study population, 2) study outcomes and endpoints | Propensity score matching, covariate adjustment |
| J Salas^19^ | 1) Initiation of other anti-diabetic medication during follow-up; 2) death and health insurance disenrollment | 1) Intention-to-treat; 2) censoring follow-up time at disenrollment, death, or end of study. | 1) Primary analysis, 2) Follow-up time | IPTW and covariate adjustment |
| Y Xu^20^ | 1) Drug exposure modification (discontinuation, addition, or switching) 2) death | 1) As-treated with induction (minimal time needed between drug initiation and disease occurrence) and latency period (maximal time between drug modification and disease occurrence); initial-treatment analysis ignoring subsequent drug use modifications as a sensitivity analysis 2) Patients were excluded if they died or modified their initial drug use within 90 days. | 1) follow-up; sensitivity analysis 2) follow-up | Stabilized IPTW |
| T Chyou^21^ | Drug discontinuation, or crossover to another antipsychotic | Censoring at the ICE | Study population | Stabilized IPTW |
| T Thai^22^ | Induced abortions, ectopic pregnancies | exclusion | Study population, design, and data source | Stabilized IPTW |
| E Skovlund^23^ | NA (exposure was categorized, only children having reached age of five years are included) | NA | NA | Potential confounder adjusted in logistic model |
| M Webster-Clark^24^ | 1) Drug discontinuation or addition 2) death for the other two outcomes | 1) Both ITT and on-treatment 2) using a weighted Aalen-Johansen estimator to take into account the competing risk of death | 1) Exposure 2) Statistical analysis | IPTW, SMR weights and IPCW |
| B Harding^25^ | death | censored | Statistical analysis | Time-varying exposure used in modeling , Covariate adjustment |
| T Althunian^26^ | 1) Drug switching/stopping 2) death | 1) Per-protocol (censored at the occurrence), ITT, As-treated (time-varying) 2) censored | Medication exposure and analysis populations | Covariate adjustment |
| L. Fanning^27^ | 1) treatment discontinuation or treatment switch (to aspirin or any anticoagulant), 2) death, transfer out of the practice (UK patients) | 1) On-treatment (censored) 2) censored | Outcome and cohort follow-up | propensity-score (PS) fine stratification |
| C Srinivas^28^ | 1) Treatment switch 2) death, emigration | 1) Ever-never exposure definition was used to handle all the drug exposures, that is, each individual's follow-up time was attributed to the drug they were first exposed to during the study period 2) censored | 1) Exposures 2) Exposures | Covariate adjustment |
| M Mahic^29^ | Emigration, death | Censored | Statistical analysis | standardized probability of treatment weighting (SPTW) based on PS |
| D Selig^30^ | 1) multiple antimuscarinic agents dispensed during follow-up 2) loss of eligibility, death | 1) the effects of duration and index drug were restricted only to patients with a single drug during follow-up. 2) censored | Statistical analysis | Covariate adjustment |
| A Gilsenan^31^ | 1) death 2) Overlaps or gaps in drug dispensing. | 1) censored 2) ignored | 1) Study design 2) Results (Footnote of Table 3) | Exposed patients matched for age, sex, three-digit zip code, date of claim for filled prescription, and number of unique therapeutic classes of medications dispensed in the previous 4 months. |
| D Barthold^32^ | 1) Using both NSL and M3S BAMs 2) Death or switch into Medicare Advantage | 1) included a sensitivity analysis with the sample restricted to individuals who only used one type of BAM 2) In a sensitivity analysis excluding those patients | Discussion | multivariable logistic regression |
| L Pasina^33^ | NA (as the study analyzed only the data collected at the baseline of the cohort study) | NA | NA | multivariable linear and logistic regression |
| D Sato^34^ | NA due to short-term low dose exposure within 7 days of surgery | NA | NA | one-to-one propensity score-matched analyses |

IPTW- inverse probability of treatment weighting, SMR- standardized mortality weighting, IPCW – inverse probability of censoring weighting

Supplemental Table 2A. Case-Control studies and features of estimand attributes including exposure, target population and outcome.

| **First Author and Reference** | **Study Question** | **Data Type/source** | **Confounder**  **control** | **Exposure** | **Target population of interest** | **Outcome measure of interest** | **Population-level Summary** |
| --- | --- | --- | --- | --- | --- | --- | --- |
| J. Busyby^10^ | Investigation into the association between 5AR inhibitor use and gastro-oesophageal cancer risk | Primary Care Clinical Information Unit Research (PCCIUR) database, Scotland/ EHR | Matching, sensitivity analysis using multiple imputation, negative control analysis | 5α‐reductase (AR) inhibitors user | Male 5α‐reductase (AR) inhibitors users | Male gastro‐oesophageal cancer cases matched to up to 5 controls | Odds Ratio |
| L.C. Saiz^11^ | To examine the association between use of methylphenidate and the risk for valvular heart disease (VHD) | Primary care database (BIFAP), Spain/EHR | 1:10 variable ratio matching, multivariable conditional logistic regression | Methylphenidate use | Patients aged 5 to 25 years registered in BIFAP during 1 January 2002 to 31 December 2014. | valvular heart disease (VHD) | Odds Ratio |
| Y. Miyamoto^12*^ | To investigate the association between the use of pregabalin and injury. | Japan Medical Data Center (JMDC) claims database, Japan/claims | 1:4 Matching on age, sex, calendar day of injury and follow-up duration; self-control in case-crossover design | Pregabalin use | Patients aged≥20 years who had been registered for ≥6 months prior to the study period 2014-2016 | Injury | Odds Ratio |
| A Saad^13^ | This study was undertaken to evaluate the effect of beta-blockers on pancreatic ductal adenocarcinoma (PDAC) risk according to receptor selectivity and treatment duration. | The Health Improvement Network UK /EHR | Variable ratio matching on age at index-date, sex, practice site, and both duration and calendar time of follow-up. Covariate adjustment in conditional logistic regression | Beta-blocker, (users vs. never users; selective vs. non-selective beta-blocker, active vs. former users) | Individuals receiving medical care from a THIN practitioner between 1995 and 2013. | Incident diagnosis of PDAC during follow-up. | Odds Ratio |
| J Lai^14^ | To evaluate whether concomitant use of amiodarone and sulfonylureas is associated with an increased risk of serious hypoglycemia. | Health Insurance database, Taiwan/claims | Up to 1:10 matching on age, sex, cohort entry date, four types of individual sulfonylureas, and DM therapy regimen | concomitant use of amiodarone and sulfonylureas vs sulfonylureas alone | diabetic patients aged≥20 years at the first recorded sulfonylurea prescription during the study period (January1, 2009-June 30, 2013) | severe hypoglycemia and a composite outcome of severe hypoglycemia, altered consciousness, and fall-related fracture | Odds Ratio |
| S. Shin^15**^ | To assess the association between domperidone and adverse cardiovascular events | National Health Insurance Service-National Sample Cohort (NHIS-NSC), South Korea/claims | 1:2 case control matching and covariate adjustment in modeling | exposure to domperidone compared to exposure to metoclopramide, or none of the two | patients without a history of hospitalizations, cancer, or cardiovascular diseases | a composite endpoint comprised of arrhythmia and acute myocardial infarction | Odds Ratio |

***** Nested case-control and case-crossover ** nested case-control and case-time-control study

Supplemental Table 2B. Case-Control studies and details of case and exposure definitions.

| **Reference** | **Cohort definition** | **Details of exposure definition** | **Covariates/terminal events (potential ICEs)** |
| --- | --- | --- | --- |
| J. Busyby^10^ | Between 1993 and 2011, the PCCIUR collected computerised medical records from approximately 15% of the Scottish general practice population and included details on patient demographics (eg, age and gender), primary care encounters, clinical diagnoses, and prescriptions. | we extracted data on the medication prescribed, number of packs/tablets and medication strength, and calculated defined daily doses (DDDs). A single DDD is the average maintenance dose per day of a drug used for its main indication in adults (eg, BPH for finasteride). We calculated the total number of DDDs received during the exposure window and categorised patients into those receiving 0 (never users), between 1 and 365 DDDs (less than 1 year of usage), between 366 and 1095 DDDs (between 1 and 3 years of usage), and more than 1095 (more than 3 years of usage) | Cases and controls with an earlier cancer diagnosis (other than nonmelanoma skin cancer), and those with less than a 3-year exposure assessment window were excluded from the study |
| L.C. Saiz^11^ | The study cohort included those patients aged 5 to 25 years registered with the primary care physicians (PCPs) in the study period (1 January 2002 and 31 December 2014) and with at least 1-year registry with their PCP. People entered the study cohort (start date) once they fulfilled all of the previous inclusion criteria. | Patients were classified as current users when there was a record of exposure to methylphenidate in the 3 months prior to the index date; recent users when exposure ended between 3 and 12 months before the index date; past users when exposure ended more than 12 months before the index date; and never users, when there was no evidence of exposure to methylphenidate prior to the index date | Patients included in the study cohort were followed until the earliest of the following events: occurrence of the event of interest (VHD), 26-years old, diagnosis of other cancer, death, lost to follow-up, or end of the study period. |
| Y. Miyamoto^12*^ | The JMDC claims database includes health insurance claims data on outpatient, inpatient, and pharmacy for employed workers in medium- to large-scale companies and their family members from 2005 to 2016, during which the cumulative observed population was 4 million. The study population comprised people aged ≥20 years who had been registered for ≥6 months and contributed to the database after January 2014 (because cases were identified after January 2014, as explained below). | The primary analysis defined exposure as at least one prescription of pregabalin within 180 days prior to index date. In the sensitivity analysis #1, the 180 days was changed to 90 days. In the sensitivity analysis #2, people were categorized into 3 groups: within 30 days, 31 to 90 days, and 91 to 180 days before the index date. | We also considered drugs with anticholinergic properties other than the above-specified drugs, and the drugs related to bone density. The Charlson Comorbidity Index was calculated for baseline comorbidities (congestive heart failure, dementia, chronic pulmonary disease, rheumatic disease, mild liver disease, diabetes with chronic complications, hemiplegia or paraplegia, renal disease, any malignancy without metastasis, moderate or severe liver disease, metastatic solid tumor, or acquired immunodeficiency syndrome) using relevant diagnoses recorded at any time point from 180 days before the index date until the index date. We also considered a history of cardiac arrhythmia, dementia, and alcohol abuse within 180 days before the index date |
| A Saad^13^ | Individuals receiving medical care from a THIN practitioner between 1995 and 2013 were eligible for inclusion in the study.26 Follow-up time started at the later of either the date when the THIN practice started using the electronic medical record software or 6 months after the date at which the patient registered with the clinic.27 Follow-up time ended on the PDAC diagnosis date for cases (index date) and on the same assigned date for the matched controls. | The primary exposure of interest was defined as any prescription with beta-blockers prior to index date. Beta-blockers use was further stratified to non-selective beta-blockers that display both β1- and β2-antagonism (propranolol, labetalol, carvedilol, sotalol, carteolol, nadolol) and β1-selective blockers (bisoprolol, atenolol, metoprolol, celiprolol, betaxolol, acebutolol, nebivolol). Beta-blocker users were further classified as active users and former users. Active users were defined and stratified for analysis as individuals with their last prescription within 6 months prior to PDAC diagnosis and first prescription either 0 to 1 year, 1 to 2 years or more than 2 years prior to index date. Former users were defined as individuals with last prescription more than 6 months prior to PDAC diagnosis | Covariates selected include; obesity defined as BMI above 30 kg/m2, smoking defined as ever/never, alcohol consumption, past medical history of diabetes mellitus, cardiovascular disease, hypertension (based on diagnostic Read codes), or any calcium channel blocker (CCB) use prior to the index date. |
| J Lai^14^ | We defined the cohort entry date as the first recorded prescription for a sulfonylurea during the study period, and then we followed patients from that date until the earliest of the following events: occurrence of hypoglycemic outcomes (defined below), disenrollment of NHI, discontinuous use of sulfonylureas, death, and the end of the study period. | The use of amiodarone was classified into current use (1-30 days), recent use (31-180 days), past use (181-365 days), and remote use (>365 days) according to the end of the most recent prescription of amiodarone before the index date. | We considered multiple demographic and clinical characteristics that may associate with amiodarone use or occurrence of hypoglycemia. Age, sex, and proxy indicators of diabetic severity, such as dose of sulfonylureas, number of hemoglobin A1C (HbA1C) test, and complexity of DM therapy regimen were measured in the year preceding the index date. We also measured diabetic complications, comorbidities that may lead to dysglycemia, such as coronary artery disease, chronic kidney disease, liver disease, and sepsis, and comorbid conditions that may result from adverse events with amiodarone use, including thyroid dysfunction, pulmonary fibrosis, and acute pancreatitis, which were measured in the year prior to the index date (outcome date or matched control date). Number of insulin prescriptions, medications that may cause dysglycemia, such as corticosteroids, angiotensin converting enzyme inhibitors, and thiazide diuretics, rate control medications, antidiabetic medications, and medications that may influence the CYP450 enzyme system were additionally examined in the 180 days before the index date. |
| S. Shin^15**^ | From all patients included in the NHIS-NSC database, we identified patients without a history of hospitalizations, cancer, or cardiovascular diseases in 2002, as baseline characteristics were assessed in the year 2002. Cohort entry was defined as 1 January 2003 for all patients included in the study. We excluded the following patients from our study: (a) those hospitalized for any cause or diagnosed with cancer within the year before cohort entry as such severe conditions may affect patient's subsequent cardiac conditions; (b) those diagnosed with cardiovascular events in 2002 to restrict to incident cardiovascular events and not recurrent | We used both inpatient and outpatient prescription records to define exposure to domperidone and metoclopramide. The exposure ascertainment period was defined as the 7-day window prior to the index date for cases and controls. All patients were classified into discrete categories of domperidone use, metoclopramide use, or non-use of both drugs. | After matching, we further excluded all individuals who received palliative care (Z51.5) or ≥ 2 potent opioid analgesic prescriptions within 45 days prior to the index date, where the case-control pair were excluded |

***** Nested case-control and case-crossover ** nested case-control and case-time-control study
